# Pre-transplant dialysis vintage and post-transplant outcomes: A retrospective cohort study in Korean kidney transplant recipients

**DOI:** 10.1371/journal.pone.0352995

**Published:** 2026-07-20

**Authors:** Eunju Jang, Ki Yoon Moon, Sang Seob Yun, Sun Cheol Park

**Affiliations:** Division of Vascular and Transplant Surgery, Department of Surgery, Seoul St. Mary’s Hospital, College of Medicine, The Catholic University of Korea, Seoul, Republic of Korea; Showa University, JAPAN

## Abstract

**Background:**

Kidney transplantation (KT) is the preferred treatment for end-stage renal disease (ESRD) due to its advantages over dialysis. Although the benefits of preemptive KT are well established, the association between pre-transplant dialysis duration and post-transplant outcomes remains uncertain.

**Objective:**

To evaluate the association between pre-transplant dialysis vintage and post-transplant outcomes in Korean KT recipients using real-world data from a tertiary transplant center.

**Methods:**

We retrospectively evaluated 1,843 first KT recipients transplanted between 2006–2021. Dialysis duration was used as a categorical variable divided by tertiles according to distribution of time of analysis, and preemptive transplantation was categorized as a separate group. Primary outcomes included death-censored graft loss, all-cause mortality and composite outcomes. Survival was assessed using Kaplan–Meier and Cox proportional hazards models at a 95% confidence level. A deceased-donor KT (DDKT) subgroup analysis was also performed.

**Results:**

369 patients underwent preemptive KT, and 1,474 dialysis-exposed recipients were categorized into tertile 1 (n = 492), tertile 2 (n = 491), and tertile 3 (n = 491), with mean pre-transplant dialysis durations of 1.2, 21.4, and 107.6 months, respectively. Compared with preemptive KT, all-cause mortality did not differ in the first or second tertiles but was significantly higher in the third tertile (aHR 3.55; 95% CI 1.43–8.81; *p* = 0.006). In the overall cohort, the third tertile was also associated with higher risks of death-censored graft failure and the composite outcome. However, in the deceased-donor KT subgroup, death-censored graft failure did not differ significantly across tertiles, and the composite outcome did not show a monotonic dose-response pattern.

**Conclusions:**

Prolonged pre-transplant dialysis was associated with a higher risk of all-cause mortality, whereas its association with graft failure was less consistent, particularly among deceased-donor KT recipients.

## Introduction

Kidney transplantation (KT) is the treatment of choice for selected patients with ESRD and is associated with improved patient survival and quality of life compared with dialysis [1–3]. However, the waiting list for renal transplantation is larger than the availability of organs. In South Korea, the total number of patients being enrolled for kidney transplantation has continued to increase from 12,463–31,055 patients over the past 10 years (from 2012 to 2021), while annual kidney transplantation cases from living and deceased donors remain at an average of 840 per year, with a slightly decreasing trend in recent 5 years [4].

Evidence for prolonged survival for kidney transplant recipients compared with patients on hemodialysis or peritoneal dialysis has long been established [5,6]. Also, dialysis vintage, the duration of dialysis before KT, has been associated with increased risk of patient death or and graft in various studies [7–11], which underscores the need for early kidney transplantation with minimal waiting time, and preparation for preemptive transplantation before progression to end-stage renal disease (ESRD).

Although the advantage of transplantation over dialysis, and preemptive KT is well established, there have been studies questioning the direct association of dialysis vintage with allograft survival. Past studies have identified dialysis vintage as an independent risk factor for adverse effects on graft and patient survival [11,12]. Due to advancements in personalized immunosuppressive protocols and better techniques for matching donors and recipients, the success rates of transplants have significantly increased over time [13,14]. Recent data from large transplant registries indicates that survival rates on maintenance dialysis have improved, even with an older population and more advanced stages of ESRD [15,16]. Recent studies have also noted that advancements in dialysis protocols and immunosuppressant have reduced the impact of dialysis duration itself on transplantation outcomes [17,18].

Despite well-documented benefits of preemptive kidney transplantation, the influence of dialysis duration before KT on long-term graft and patient survival remains debated, particularly in Asian populations with limited donor availability. Several Korean studies have described trends in preemptive KT and the effect of dialysis vintage, especially among deceased-donor KT (DDKT) recipients. [2,19,20]. However, single-center data may provide complementary information through more uniform perioperative management, detailed clinical covariate adjustment, clear separation of dialysis-naïve preemptive KT recipients from short-term dialysis recipients and competing-risk assessment of graft failure. To complement these existing data, this study aimed to assess the impact of pre-transplant dialysis vintage on post-transplant outcomes in Korean renal transplant recipients and to provide additional real-world evidence from a tertiary transplant center.

## Materials and methods

We performed a retrospective cohort study using clinical data from adult kidney transplant recipients at Seoul St. Mary’s Hospital (Seoul, Republic of Korea) who underwent transplantation between 2006 and 2021. We included first-time, single-organ kidney transplant recipients (living- or deceased-donor) aged ≥18 years, and excluded recipients of multiple kidney transplants and simultaneous multi-organ transplantation (e.g., pancreas, liver, or heart). Among recipients who underwent dialysis before transplantation, dialysis vintage was categorized into tertiles according to its distribution in the analytic cohort: tertile 1, 0.5–4 months; tertile 2, 4–52 months; and tertile 3, 52–334 months. These tertile cutoffs were cohort-derived and used for exploratory risk stratification, rather than as predefined clinical or allocation thresholds. Preemptive transplantation was analyzed as a separate group.

This study represents a secondary analysis of data extracted under an existing Institutional Review Board (IRB)–approved protocol originally approved for a broader retrospective study on tacrolimus exposure and transplant outcomes (“Tacrolimus exposure and transplant outcomes in renal allograft recipients: A Retrospective Multicenter Study of Clinical Data Warehouse (CDW) in South Korea”). For the present analysis, only the Seoul St. Mary’s Hospital subset was used. The study was approved by the Institutional Review Board of Seoul St. Mary’s Hospital, and the requirement for informed consent was waived due to the retrospective nature of the study (IRB No. KC21WIDI0910). Data were accessed for research purposes on March 28, 2022. Data were analyzed in a de-identified format, and the study posed minimal risk to participants.

### Definitions

Pre-transplant dialysis vintage was defined as the duration from dialysis initiation to kidney transplantation. Preemptive kidney transplantation was defined as transplantation performed before the initiation of dialysis. Patients who had received either hemodialysis or peritoneal dialysis at any point before transplantation were not included in the preemptive group. Pre-transplant dialysis modality was classified as hemodialysis, peritoneal dialysis, or mixed modality for patients who switched dialysis modality before transplantation. Primary outcomes were the occurrence of death-censored graft loss, all-cause mortality and the composite of both outcomes during the study period. Patient survival time was defined as the time from transplantation to patient death or end of follow-up. Graft loss was defined as return to permanent dialysis or second renal transplantation and was censored for death.

Secondary outcomes included major adverse cardiovascular events (MACE), biopsy-proven acute rejection (BPAR) and delayed graft function (DGF). MACE included stroke or ischemic cardiovascular events leading to hospitalization, or death by cardiovascular cause. DGF was identified when dialysis was done during the first week after transplantation.

The distribution of dialysis vintage was not even between the three tertile groups, with a much shorter waiting time for LDKT recipients compared with DDKT recipients. A subgroup analysis including only DDKT recipients was performed to achieve a more balanced distribution of pretransplant dialysis duration and to eliminate the impact of differences owing to living and cadaveric donors.

### Statistical analysis

Continuous variables are presented as mean  ±  standard deviation or median (interquartile range; IQR) depending on their distribution, and categorical variables are presented as number and percentage. One-way analysis of variance or the Kruskal–Wallis test was used to determine the differences in continuous variables, as appropriate, whereas for categorical variables, Pearson’s chi-square test or Fisher’s exact test was used. Kaplan–Meier curves and the log-rank test were used to compare the differences in graft survival, patient survival and composite event-free survival. The association between pretransplant dialysis vintage and clinical outcomes, including composite outcomes, was further determined using multivariable Cox proportional hazard regression models. The adjustment factors selected were baseline characteristics and clinically relevant variables. Patient death can influence graft failure as a competing event. Therefore, we employed the Fine and Gray competing risk model to account for patient death and compare the risk of graft failure. Subgroup analyses by age, sex, body mass index, comorbidities, DDKT, desensitization, induction agent, transplantation year, donor age and BMI were performed for patient death and graft failure. Graft failure was also analyzed by competing risk analysis in subgroup analysis. Statistical analyses were performed with SPSS version 22.0 (IBM Corp., Armonk, NY, USA) and R (R Foundation for Statistical Computing, Vienna, Austria; www.r-project.org). *P* values less than 0.05 were considered statistically significant.

### Results

**Fig 1:**
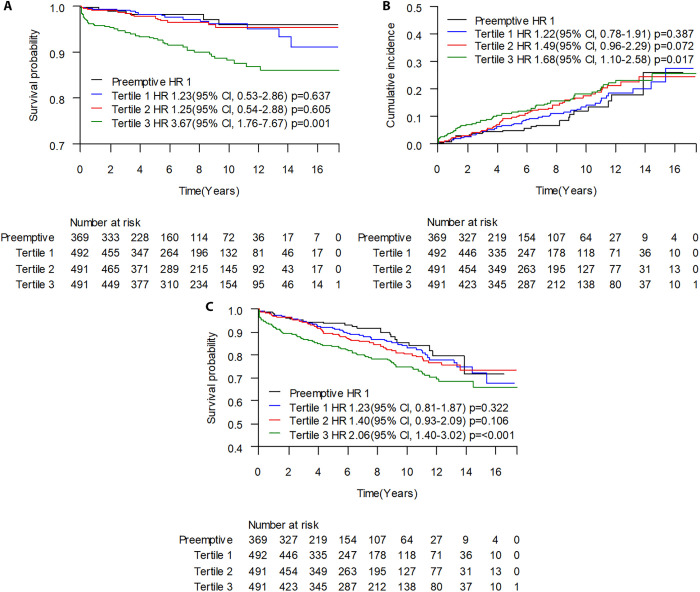
Kaplan-Meier curves for primary outcomes. **(A)** All-cause mortality, **(B)** Graft failure, **(C)** Composite outcome.

### Baseline characteristics

Total 1843 first KT recipients were included in our analysis. The baseline characteristics of the preemptive group and three groups stratified by pretransplant dialysis vintage is shown in Table 1. The preemptive group included 369 patients, all of whom received living donor kidney transplantation except two. The first tertile included 492 patients with mean pretransplant dialysis period of 1.2 months, second tertile included 491 patients with mean pretransplant dialysis period of 21.4 months, and the third tertile also included 491 patients with mean pretransplant dialysis period of 107.6 months. The proportion of DDKT was significantly higher with longer pretransplant dialysis duration increasing from 1.2% in the first tertile, to 20.6% in the second tertile, and 80.4% in the third tertile (*p* < 0.001). Consequently, the use of antithymocyte globulin (ATG) as induction agent, and duration of total ischemic time was highest in the third tertile group (32.8% and 166.2 minutes, *p* < 0.001).

**Table 1:** Baseline characteristics of study patients.

	Total (n = 1843)	Preemptive (n = 369)	Tertile 1 (n = 492)	Tertile 2 (n = 491)	Tertile 3 (n = 491)	*p*-value
**Recipient age (year)**	47.0 ± 11.8	46.5 ± 12.1	44.6 ± 12.7	47.2 ± 11.9	49.8 ± 9.8	<0.001
**Recipient sex (n, %)**						0.419
**Male**	1075 (58.3)	212 (57.5)	297 (60.4)	293 (59.7)	273 (55.6)	
**Female**	768 (41.7)	157 (42.5)	195 (39.6)	198 (40.3)	218 (44.4)	
**Recipient BMI (kg/m**^**2**^)	23.2 ± 3.6	23.1 ± 3.7	23.2 ± 3.7	23.3 ± 3.6	23.1 ± 3.4	0.672
**DM (n, %)**	530 (28.8)	105 (28.5)	155 (31.5)	166 (33.8)	104 (21.2)	<0.001
**HTN (n, %)**	1560 (84.6)	320 (86.7)	425 (86.4)	404 (82.3)	411 (83.7)	0.186
**Primary renal diagnosis** **(n, %)**						<0.001
**DM**	441 (23.9)	90 (24.4)	130 (26.4)	139 (28.3)	82 (16.7)	
**HTN**	287 (15.6)	33 (8.9)	53 (10.8)	79 (16.1)	122 (24.8)	
**Glomerulonephritis**	588 (31.9)	129 (35.0)	170 (34.6)	129 (26.3)	160 (32.6)	
**Polycystic kidney disease**	88 (4.8)	20 (5.4)	20 (4.1)	25 (5.1)	23 (4.7)	
**Others**	108 (5.9)	24 (6.5)	25 (5.1)	36 (7.3)	23 (4.7)	
**Unknown**	327 (17.7)	70 (19.0)	94 (19.1)	82 (16.7)	81 (16.5)	
**Missing**	4 (0.2)	3 (0.8)	0 (0.0)	1 (0.2)	0 (0.0)	
**Pretransplant dialysis duration (month)**	34.7 ± 52.4	0.0 ± 0.0	1.2 ± 1.1	21.4 ± 14.5	107.6 ± 50.9	NA
**Dialysis modality**	n = 1474					<0.001
**HD**	1125 (76.3)	NA	474 (96.3)	360 (73.3)	291 (59.3)	
**PD**	245 (16.6)	NA	12 (2.4)	101 (20.6)	132 (26.9)	
**Mixed**	104 (7.1)	NA	6 (1.2)	30 (6.1)	68 (13.8)	
**DDKT (n, %)**	504 (27.3)	2 (0.5)	6 (1.2)	101 (20.6)	395 (80.4)	<0.001
**Sum of HLA mismatch number (Median, IQR)**	3 (2-5)	3 (2-5)	3 (2-4)	3 (2-4)	4 (3-5)	<0.001
**Desensitization (n, %)**	568 (30.8)	145 (39.3)	188 (38.2)	160 (32.6)	75 (15.3)	<0.001
**Plasmapheresis (times)**	1.1 ± 2.3	1.5 ± 2.6	1.3 ± 2.3	1.3 ± 2.6	0.3 ± 1.4	<0.001
**Induction immunosuppression (n, %)**						<0.001
**Basiliximab**	1457 (79.1)	307 (83.2)	425 (86.4)	395 (80.4)	330 (67.2)	
**Anti-thymocyte globulin**	386 (20.9)	62 (16.8)	67 (13.6)	96 (19.6)	161 (32.8)	
**Total ischemic time (min)**	95.1 ± 86.2	57.7 ± 24.3	57.3 ± 41.7	90.5 ± 85.7	166.2 ± 104.5	<0.001
**Donor age (year)**	45.0 ± 12.7	45.6 ± 12.3	44.5 ± 12.4	44.8 ± 12.7	45.2 ± 13.3	0.446
**Donor BMI (kg/m**^**2**^)	23.9 ± 3.5	24.0 ± 3.3	23.9 ± 3.3	23.9 ± 3.5	24.0 ± 3.9	0.952

*p*-values were calculated using the chi-square, Fisher's exact test or Kruskal–Wallis tests. BMI, body mass index; DM, diabetes mellitus; HTN, hypertension; HD, hemodialysis; PD, peritoneal dialysis; DDKT, deceased donor kidney transplant; HLA, human leukocyte antigen; IQR, interquartile range; NA, not applicable.

The primary outcomes in each study group are summarized in Table 2. A total of 285 composite events (15.5%) were observed. Occurrence of all-cause mortality (*p* < 0.001), graft failure (*p* = 0.001) and the composite outcomes (*p* < 0.001) were all significantly higher with the increase of pretransplant dialysis duration. The rate of biopsy-proven acute rejection (20.7%), delayed graft function (5.1%) and major cardiovascular events (6.6%) were also observed as secondary outcomes. There were no significant differences between groups for BPAR (*p* = 0.821), but DGF (*p* < 0.001) and cardiovascular events (*p* = 0.001) were significantly higher with longer pretransplant dialysis periods.

**Table 2:** Outcomes after kidney transplantation.

	Total (n = 1843)	Preemptive (n = 369)	Tertile 1 (n = 492)	Tertile 2 (n = 491)	Tertile 3 (n = 491)	*p*-value
**All-cause mortality (n, %)**	88 (4.8)	8 (2.2)	15 (3.0)	16 (3.3)	49 (10.0)	<0.001
**Overall survival (months)**	87.6 ± 50.8	74.9 ± 46.7	86.7 ± 52.0	91.9 ± 50.5	93.7 ± 51.4	<0.001
**Graft failure (n, %)**	232 (12.6)	29 (7.9)	54 (11.0)	68 (13.8)	81 (16.5)	0.001
**Graft survival (months)**	81.8 ± 50.2	71.2 ± 44.8	81.9 ± 50.6	85.4 ± 50.1	86.2 ± 52.8	<0.001
**Composite outcome (n, %)**	285 (15.5)	34 (9.2)	64 (13.0)	75 (15.3)	112 (22.8)	<0.001
**BPAR (n, %)**	382 (20.7)	71 (19.2)	100 (20.3)	106 (21.6)	105 (21.4)	0.827
**DGF (n, %)**	94 (5.1)	1 (0.3)	6 (1.2)	23 (4.7)	64 (13.0)	<0.001
**CVE (n, %)**	121 (6.6)	15 (4.1)	24 (4.9)	32 (6.5)	50 (10.2)	0.001

*p*-values were calculated using the chi-square, Fisher's exact test or Kruskal–Wallis tests. BPAR, biopsy-proven acute rejection; DGF, delayed graft function; CVE, cardiovascular event.

### All-cause mortality

Overall, 88 deaths (4.8%) occurred within the study period (Table 2). Although occurrence of mortality events was significantly higher in the higher tertile groups, the overall survival period after transplantation in each group was significantly longer in the groups with longer dialysis duration. Infection was the leading cause of death involving 33 patients (37.5%), followed by newly developed malignancy which was found in 22 cases (25.0%). Other cases of mortality were as follows; cardiovascular events (14 patients, 15.9%), acute respiratory distress syndrome (ARDS; 7 patients, 8.0%), bleeding (2 patients, 2.3%), others (5 patients, 5.7%) and unknown (5 patients, 5.7%).

Kaplan-Meier curve showed that compared with the preemptive group, risk of all-cause mortality in tertile 3 was significantly higher (hazard ratio [HR] =3.67, 95% confidence interval [CI] =1.76–7.67, *p* = 0.001) whereas the difference was not significant in tertiles 1 and 2 (Fig 1A). After accounting for confounding factors, risk of all-cause mortality was consistently higher in only the third tertile (model 3; adjusted hazard ratio [aHR] 3.55, 95% CI 1.43–8.81, *p* = 0.006) (Table 3). Older age of the recipient and male sex were independent risk factors for mortality in the multivariable Cox regression model.

**Table 3:** Cox regression analysis of all-cause mortality.

	Univariate analysis		Model 1		Model 2		Model 3	
	HR (95% CI)	*p*-value	aHR (95% CI)	*p*-value	aHR (95% CI)	*p*-value	aHR (95% CI)	*p*-value
**Preemptive**	Reference							
**Tertile 1**	1.23(0.53-2.86)	0.637	1.30(0.56-3.03)	0.548	1.30(0.55-3.06)	0.545	1.27(0.54-2.99	0.589
**Tertile 2**	1.25(0.54-2.88)	0.605	1.11(0.48-2.56)	0.813	1.14(0.49-2.69)	0.759	1.13(0.48-2.66)	0.78
**Tertile 3**	3.67(1.76-7.67)	0.001	3.14(1.50-6.57)	0.002	4.12(1.71-9.95)	0.002	3.55(1.43-8.81)	0.006
**Age**	1.08(1.06-1.11)	<0.001	1.08(1.05-1.10)	<0.001	1.08(1.05-1.10)	<0.001	1.08(1.05-1.11)	<0.001
**Sex (ref: male)**	0.56(0.35-0.88)	0.012	0.59(0.37-0.94)	0.027	0.60(0.37-0.96)	0.034	0.57(0.34-0.95)	0.032
**BMI**	1.01(0.96-1.07)	0.664	0.99(0.92-1.06)	0.717	0.98(0.92-1.05)	0.631	0.95(0.88-1.02)	0.163
**DM**	1.87(1.22-2.88)	0.004			1.48(0.93-2.36)	0.095	1.52(0.93-2.48)	0.095
**HTN**	1.11(0.63-1.97)	0.711			0.76(0.42-1.39)	0.371	0.74(0.39-1.41)	0.358
**DDKT (ref. LDKT)**	2.19(1.44-3.32)	<0.001			0.80(0.43-1.50)	0.482	1.00(0.50-2.03)	0.993
**Plasmapheresis**	1.00(0.91-1.10)	0.957			1.05(0.95-1.16)	0.318	1.06(0.96-1.18)	0.212
**ATG induction**	1.75(1.06-2.88)	0.028			1.14(0.66-1.97)	0.635	0.99(0.55-1.79)	0.971
**KT year≥2010**	1.29(0.71-2.36)	0.406			0.92(0.48-1.74)	0.792	0.65(0.31-1.37)	0.257
**Donor age**	1.01(1.00-1.03)	0.113					1.00(0.98-1.02)	0.998
**Donor BMI**	1.00(0.94-1.07)	0.921					1.02(0.96-1.08)	0.568

HR, hazard ratio; CI, confidence interval; aHR, adjusted hazard ratio; BMI, body mass index; DM, diabetes mellitus; HTN, hypertension; DDKT, deceased donor kidney transplantation; LDKT, living donor kidney transplantation; ATG, anti-thymocyte globulin.

**Fig 2:**
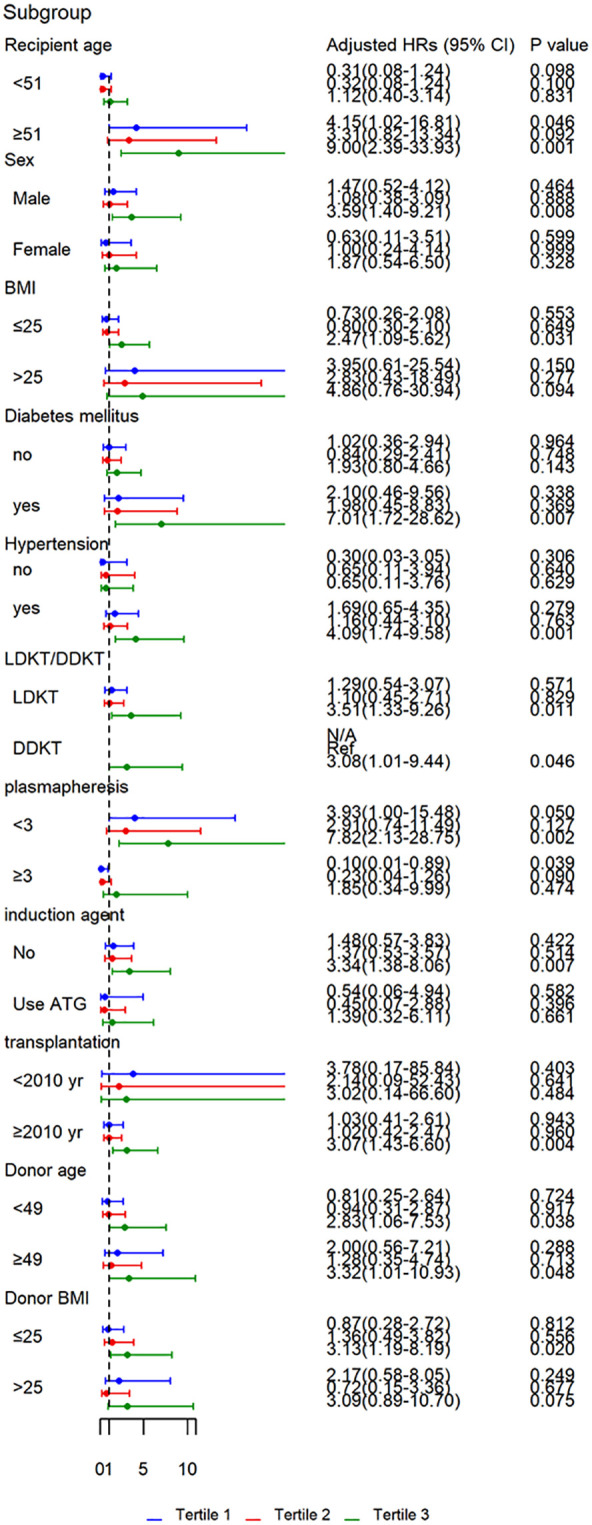
Subgroup competing risk analysis for all-cause mortality.

In the subgroup competing risk analysis for all-cause mortality, the first and second tertile group showed no increased risk of mortality in all subgroups, while the third tertile group showed an increased risk in older age group, diabetes mellitus (DM) group, hypertension (HTN) group, lower plasmapheresis group, and transplantation after 2010 groups. (Fig 2)

### Graft failure

232 recipients (12.6%) lost graft function during the study period (Table 2). The overall rate of graft failure was lowest in the preemptive group and increased with longer dialysis duration. However, the average graft survival period was longer in the groups with longer dialysis vintage; 71.2 months in the preemptive group, and 81.9 months, 85.4 months, 86.2 months respectively for tertile 1, 2 and 3. Rate of BPAR did not show a significant intergroup difference.

The Kaplan-Meier curve showed that the incidence of death-censored graft failure was significantly higher in the third tertile (HR = 1.68, 95% CI = 1.10–2.58, *p* = 0.017) compared with the preemptive group (Fig 1B). The competing risk regression model for graft failure showed that the risk for graft failure was consistently higher in the third tertile (model 3; aHR = 1.89, 95% CI = 1.05–3.40, *p* = 0.034), with pretransplant DM, plasmapheresis, operation performed before 2010, and older donor age as independent risk factors (Table 4).

**Table 4:** Competing risk regression models for graft failure.

	Univariate analysis		Model 1		Model 2		Model 3	
	HR (95% CI)	*p*-value	aHR (95% CI)	*p*-value	aHR (95% CI)	*p*-value	aHR (95% CI)	*p*-value
**Preemptive**	Reference							
**Tertile 1**	1.22(0.78-1.91)	0.387	1.21(0.77-1.90)	0.404	1.17(0.74-1.84)	0.506	1.21(0.76-1.93)	0.412
**Tertile 2**	1.49(0.96-2.29)	0.072	1.48(0.96-2.28)	0.075	1.39(0.89-2.17)	0.146	1.45(0.92-2.28)	0.106
**Tertile 3**	1.68(1.10-2.58)	0.017	1.70(1.11-2.61)	0.014	1.78(1.02-3.13)	0.043	1.89(1.05-3.40)	0.034
**Age**	1.00(0.99-1.01)	0.786	1.00(0.99-1.01)	0.702	0.99(0.98-1.00)	0.169	0.99(0.98-1.00)	0.121
**Sex (ref: male)**	0.90(0.69-1.17)	0.436	0.92(0.71-1.20)	0.559	0.90(0.69-1.17)	0.429	0.96(0.73-1.27)	0.781
**BMI**	1.03(0.99-1.07)	0.130	1.03(0.99-1.07)	0.144	1.02(0.98-1.06)	0.378	1.02(0.97-1.06)	0.427
**DM**	1.69(1.29-2.21)	<0.001			1.84(1.37-2.49)	<0.001	1.89(1.38-2.59)	<0.001
**HTN**	0.99(0.71-1.39)	0.977			0.99(0.70-1.42)	0.975	1.09(0.74-1.62)	0.659
**DDKT (ref. LDKT)**	1.28(0.97-1.69)	0.077			1.10(0.70-1.72)	0.683	1.13(0.68-1.87)	0.64
**Plasmapheresis**	1.04(0.98-1.10)	0.236			1.07(1.00-1.14)	0.044	1.07(1.00-1.14)	0.036
**ATG induction**	1.11(0.78-1.58)	0.552			1.07(0.72-1.59)	0.728	1.01(0.66-1.54)	0.96
**KT year≥2010**	0.71(0.53-0.95)	0.023			0.66(0.49-0.91)	0.010	0.59(0.41-0.85)	0.005
**Donor age**	1.02(1.01-1.03)	0.002					1.02(1.00-1.03)	0.014
**Donor BMI**	1.01(0.97-1.05)	0.607					1.01(0.97-1.05)	0.709

HR, hazard ratio; CI, confidence interval; aHR, adjusted hazard ratio; BMI, body mass index; DM, diabetes mellitus; HTN, hypertension; DDKT, deceased donor kidney transplantation; LDKT, living donor kidney transplantation; ATG, anti-thymocyte globulin.

**Fig 3:**
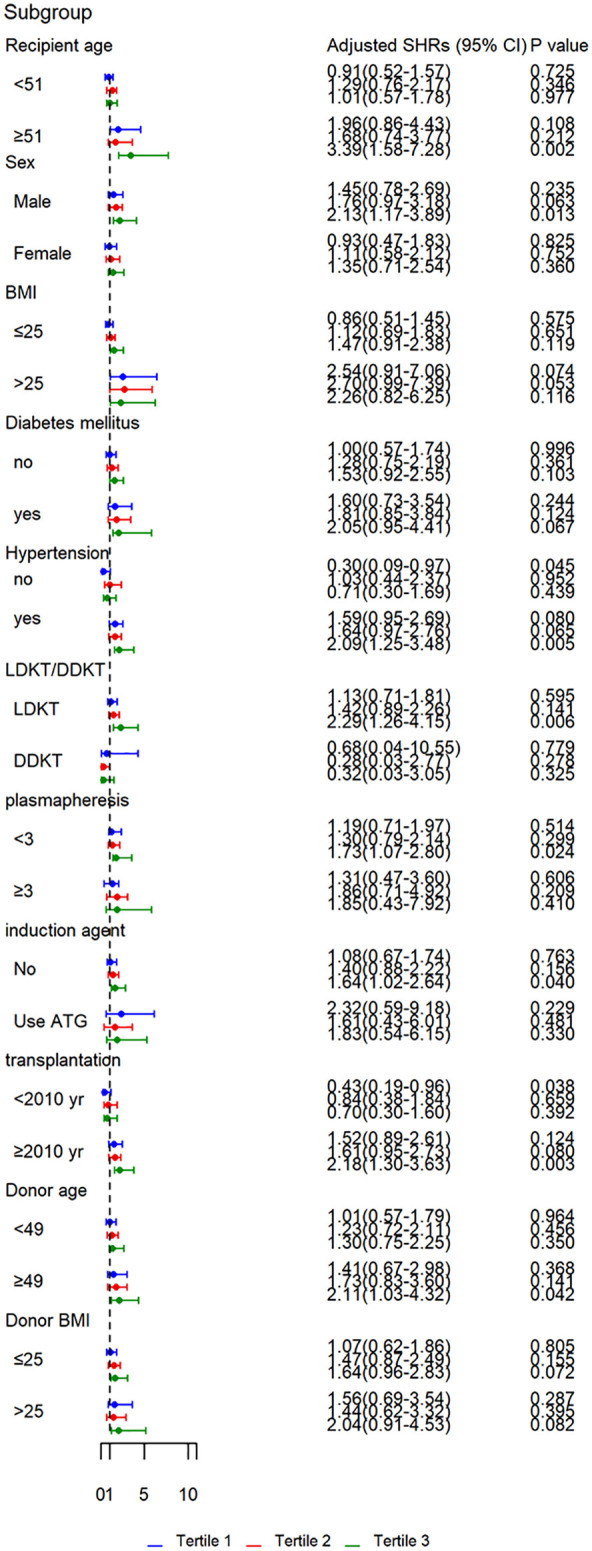
Subgroup competing risk analysis for graft failure.

In the subgroup competing risk analysis for graft failure, the first and second tertile group showed no increased risk of graft failure in all subgroup, while the third tertile group showed an increased risk in older age group, male group, DM group, HTN group, LDKT group, lower plasmapheresis group, transplantation after 2010 group, and older donor age group. (Fig 3)

### Composite outcome of both patient death and graft failure

A total of 285 (15.5%) composite events were observed during the study period (Table 2). In the Kaplan-Meier curve for the primary composite outcome, only the third tertile was significantly associated with higher risk (HR 2.06, 95% CI = 1.40–3.02, p ≤ 0.001) compared with the preemptive group (Fig 1C). Results from the multivariate Cox regression analysis showed that the third tertile group showed a significantly higher risk of composite event (model 3; aHR 2.29, 95% CI = 1.39–3.76, *p* = 0.001) (Table 5). DM, plasmapheresis, transplantation performed before 2010, and older donor age were identified as independent risk factors in the multivariate analysis.

**Table 5:** Cox regression analysis of composite outcome (all-cause mortality and death-censored graft failure).

	Univariate analysis		Model 1		Model 2		Model 3	
	HR (95% CI)	*p*-value	aHR (95% CI)	*p*-value	aHR (95% CI)	*p*-value	aHR (95% CI)	*p*-value
**Preemptive**	Reference							
**Tertile 1**	1.23(0.81-1.87)	0.322	1.25(0.83-1.90)	0.286	1.22(0.80-1.85)	0.353	1.26(0.82-1.92)	0.286
**Tertile 2**	1.40(0.93-2.09)	0.106	1.36(0.91-2.04)	0.135	1.32(0.88-2.00)	0.183	1.36(0.89-2.06)	0.155
**Tertile 3**	2.06(1.40-3.02)	<0.001	1.99(1.35-2.92)	<0.001	2.24(1.39-3.61)	0.001	2.29(1.39-3.76)	0.001
**Age**	1.02(1.01-1.03)	0.001	1.01(1.00-1.02)	0.019	1.01(1.00-1.02)	0.253	1.01(0.99-1.02)	0.248
**Sex (ref: male)**	0.81(0.64-1.03)	0.089	0.84(0.66-1.07)	0.16	0.82(0.64-1.05)	0.115	0.83(0.64-1.08)	0.165
**BMI**	1.03(1.00-1.06)	0.097	1.02(0.99-1.05)	0.272	1.01(0.98-1.05)	0.559	1.00(0.97-1.04)	0.925
**DM**	1.67(1.31-2.13)	<0.001			1.66(1.27-2.17)	<0.001	1.65(1.24-2.18)	0.001
**HTN**	1.15(0.83-1.57)	0.398			1.08(0.77-1.50)	0.663	1.19(0.83-1.71)	0.349
**DDKT (ref. LDKT)**	1.46(1.14-1.85)	0.002			0.97(0.67-1.41)	0.882	1.05(0.69-1.59)	0.836
**Plasmapheresis**	1.03(0.98-1.08)	0.226			1.06(1.01-1.12)	0.031	1.06(1.01-1.12)	0.020
**ATG induction**	1.33(0.98-1.81)	0.063			1.18(0.85-1.64)	0.325	1.08(0.76-1.53)	0.675
**KT year≥2010**	0.81(0.60-1.08)	0.15			0.70(0.51-0.95)	0.022	0.59(0.41-0.85)	0.004
**Donor age**	1.02(1.01-1.03)	<0.001					1.01(1.00-1.02)	0.020
**Donor BMI**	1.02(0.99-1.06)	0.189					1.02(0.99-1.06)	0.194

HR, hazard ratio; CI, confidence interval; aHR, adjusted hazard ratio; BMI, body mass index; DM, diabetes mellitus; HTN, hypertension; DDKT, deceased donor kidney transplantation; LDKT, living donor kidney transplantation; ATG, anti-thymocyte globulin.

**Fig 4:**
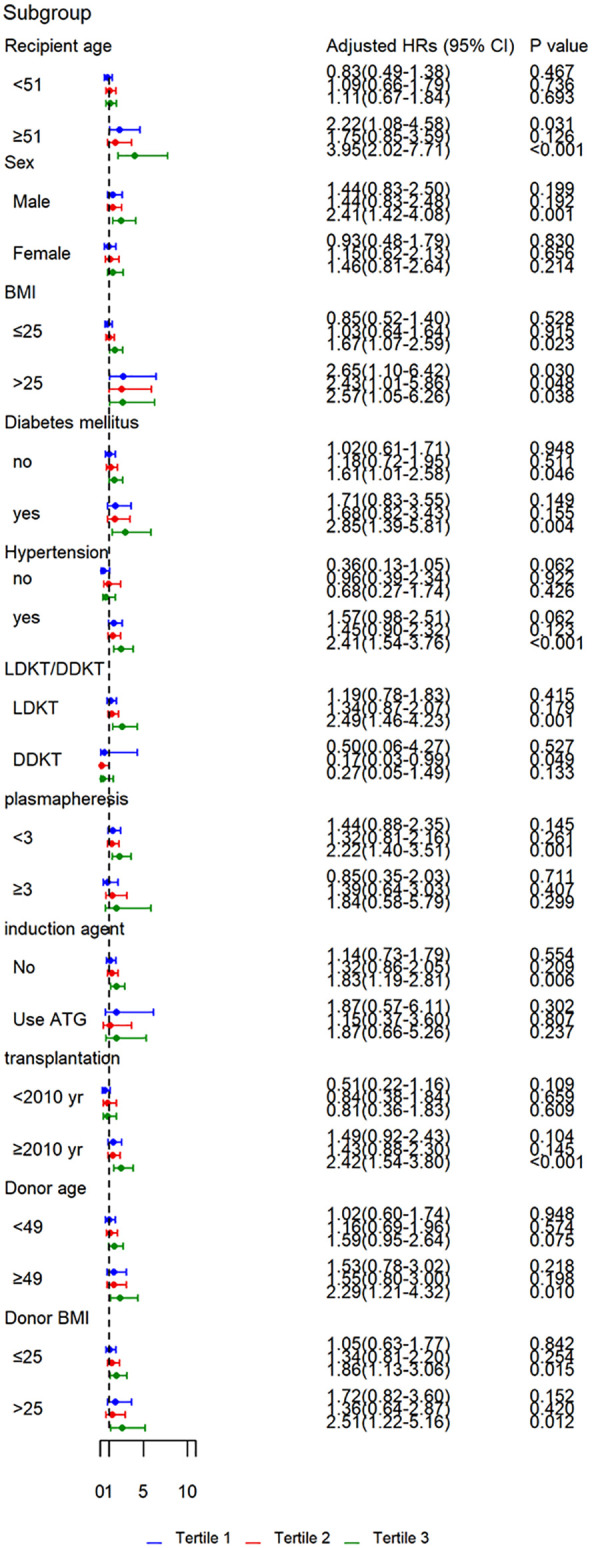
Subgroup competing risk analysis for composite outcomes.

In the subgroup competing risk analysis for composite outcomes, the first and second tertile group showed no increased risk in most subgroups, while the third tertile group showed an increased risk in older age group, male group, DM group, HTN group, LDKT group, lower plasmapheresis group, and transplantation after 2010 groups. (Fig 4)

### DDKT subgroup analysis

Due to the disproportion in DDKT, and LDKT in each tertile group, we performed a Cox regression analysis of primary outcomes involving only DDKT patients. Total 504 patients were included in the subanalysis, and evenly divided into tertiles of 168, in order of pretransplant dialysis duration. The outcomes are summarized in Tables 6–8. The Cox regression analysis for all-cause mortality in the DDKT subgroup showed a significant increase in the third tertile (Model 3; third tertile: aHR 3.25, 95% CI 1.13–9.31, p = 0.028), with age (p = 0.002) and DM (p = 0.026) as significant risk factors. The Cox regression analysis for graft failure in the DDKT subgroup showed no significant difference between the tertile groups, with donor age (aHR 1.03, 95% CI 1.00–1.05, p = 0.028) as a significant risk factor. However, the Cox regression analysis for the composite outcome in the DDKT subgroup showed a significant increase in the second tertile (Model 3; second tertile: aHR 1.87, 95% CI 1.07–3.28, p = 0.028), while the risk in the third tertile was not statistically significant.

**Table 6:** Cox regression analysis of all-cause mortality in DDKT subgroup.

	Univariate analysis		Model 1		Model 2		Model 3	
	HR (95% CI)	*p*-value	aHR (95% CI)	*p*-value	aHR (95% CI)	*p*-value	aHR (95% CI)	*p*-value
**Tertile 1**	reference		reference		reference		reference	
**Tertile 2**	3.11(1.26-7.67)	0.014	3.28(1.32-8.14)	0.010	3.55(1.43-8.83)	0.006	2.68(0.96-7.51)	0.060
**Tertile 3**	3.03(1.21-7.57)	0.018	3.03(1.20-7.60)	0.019	3.45(1.36-8.75)	0.009	3.25(1.13-9.31)	0.028
**Age**	1.08(1.04-1.12)	<0.001	1.08(1.04-1.12)	<0.001	1.07(1.03-1.11)	0.001	1.07(1.02-1.12)	0.002
**Sex (ref: male)**	0.67(0.36-1.26)	0.215	0.67(0.35-1.27)	0.218	0.77(0.40-1.48)	0.436	0.69(0.32-1.50)	0.352
**BMI**	0.97(0.88-1.07)	0.526	0.96(0.87-1.06)	0.417	0.96(0.87-1.06)	0.377	0.87(0.77-0.99)	0.036
**DM**	2.40(1.29-4.46)	0.005			2.08(1.08-3.98)	0.028	2.36(1.11-5.05)	0.026
**HTN**	1.62(0.60-4.34)	0.341			1.06(0.38-2.92)	0.914	1.01(0.30-3.40)	0.983
**ATG induction**	1.45(0.77-2.74)	0.248			1.06(0.55-2.06)	0.86	0.85(0.40-1.81)	0.675
**KT year≥2010**	2.02(0.73-5.59)	0.174			1.49(0.51-4.41)	0.467	0.40(0.06-2.59)	0.338
**Donor age**	1.02(0.99-1.04)	0.186					1.00(0.97-1.03)	0.987
**Donor BMI**	1.00(0.92-1.10)	0.913					1.02(0.93-1.12)	0.651

DDKT, deceased donor kidney transplantation; HR, hazard ratio; CI, confidence interval; aHR, adjusted hazard ratio; BMI, body mass index; DM, diabetes mellitus; HTN, hypertension; ATG, anti-thymocyte globulin.

**Table 7:** Cox regression analysis of composite outcome in DDKT subgroup.

	Univariate analysis		Model 1		Model 2		Model 3	
	HR (95% CI)	*p*-value	aHR (95% CI)	*p*-value	aHR (95% CI)	*p*-value	aHR (95% CI)	*p*-value
**Tertile 1**	reference		reference		reference		reference	
**Tertile 2**	1.75(1.09-2.79)	0.019	1.79(1.12-2.86)	0.015	1.88(1.17-3.02)	0.009	1.87(1.07-3.28)	0.028
**Tertile 3**	1.21(0.73-2.02)	0.460	1.20(0.72-2.00)	0.487	1.31(0.78-2.21)	0.310	1.36(0.72-2.57)	0.349
**Age**	1.03(1.01-1.05)	0.005	1.03(1.01-1.05)	0.006	1.03(1.00-1.05)	0.022	1.03(1.00-1.05)	0.037
**Sex (ref: male)**	0.75(0.51-1.11)	0.150	0.74(0.50-1.10)	0.141	0.76(0.51-1.14)	0.185	0.68(0.42-1.13)	0.135
**BMI**	1.00(0.95-1.06)	0.955	0.99(0.94-1.05)	0.782	0.99(0.93-1.05)	0.780	0.98(0.91-1.06)	0.645
**DM**	1.64(1.09-2.46)	0.018			1.53(0.99-2.36)	0.057	1.44(0.87-2.38)	0.157
**HTN**	1.14(0.66-2.00)	0.635			0.98(0.55-1.75)	0.958	0.96(0.45-2.03)	0.907
**ATG induction**	1.08(0.71-1.63)	0.724			1.02(0.66-1.57)	0.938	0.89(0.55-1.44)	0.628
**KT year≥2010**	1.00(0.60-1.66)	0.998			0.86(0.50-1.49)	0.596	0.86(0.22-3.31)	0.831
**Donor age**	1.03(1.01-1.05)	0.001					1.02(1.00-1.04)	0.106
**Donor BMI**	1.01(0.96-1.07)	0.650					1.02(0.96-1.09)	0.471

DDKT, deceased donor kidney transplantation; HR, hazard ratio; CI, confidence interval; aHR, adjusted hazard ratio; BMI, body mass index; DM, diabetes mellitus; HTN, hypertension; ATG, anti-thymocyte globulin.

**Table 8:** Cox regression analysis of graft failure in DDKT subgroup.

	Univariate analysis		Model 1		Model 2		Model 3	
	HR (95% CI)	*p*-value	aHR (95% CI)	*p*-value	aHR (95% CI)	*p*-value	aHR (95% CI)	*p*-value
**Tertile 1**	reference		reference		reference		reference	
**Tertile 2**	1.38(0.83-2.30)	0.217	1.40(0.84-2.33)	0.192	1.43(0.86-2.38)	0.166	1.53(0.86-2.74)	0.149
**Tertile 3**	0.94(0.53-1.67)	0.838	0.93(0.53-1.66)	0.818	1.00(0.55-1.79)	0.993	0.99(0.48-2.02)	0.973
**Age**	1.02(0.99-1.04)	0.131	1.02(0.99-1.04)	0.154	1.02(0.99-1.04)	0.175	1.02(0.98-1.05)	0.334
**Sex (ref: male)**	0.74(0.48-1.16)	0.196	0.74(0.48-1.15)	0.182	0.74(0.47-1.15)	0.183	0.76(0.43-1.33)	0.337
**BMI**	1.01(0.95-1.08)	0.648	1.01(0.94-1.07)	0.857	1.01(0.94-1.07)	0.831	1.02(0.94-1.11)	0.594
**DM**	1.50(0.94-2.39)	0.092			1.42(0.86-2.34)	0.171	1.35(0.75-2.45)	0.317
**HTN**	1.01(0.54-1.89)	0.980			0.89(0.47-1.70)	0.729	0.83(0.37-1.86)	0.647
**ATG induction**	0.87(0.53-1.41)	0.570			0.86(0.51-1.44)	0.563	0.78(0.45-1.34)	0.363
**KT year≥2010**	0.87(0.52-1.46)	0.590			0.79(0.45-1.37)	0.403	1.78(0.23-13.97)	0.585
**Donor age**	1.03(1.01-1.05)	0.003					1.03(1.00-1.05)	0.028
**Donor BMI**	0.98(0.92-1.05)	0.627					0.99(0.91-1.07)	0.766

DDKT, deceased donor kidney transplantation; HR, hazard ratio; CI, confidence interval; aHR, adjusted hazard ratio; BMI, body mass index; DM, diabetes mellitus; HTN, hypertension; ATG, anti-thymocyte globulin.

## Discussion

This study demonstrated that a longer pretransplant dialysis is associated with a higher all-cause mortality, and composite outcome of all-cause mortality and death-censored graft loss. However, the risk was not directly proportional to the duration of pretransplant dialysis. The third tertile group showed a significantly higher risk of all-cause mortality, whereas the risks in the first and second tertile groups were not significantly different from those in the preemptive group.

We had a sufficiently large sample of preemptive transplants to treat them as a distinct group and ensured that this group consisted of patients who had no dialysis before transplantation. This allowed us to clearly distinguish the impact of preemptive transplantation from short-term dialysis. The beneficial effects of preemptive KT have been well established by previous studies, in which preemptive transplantation was associated with improved transplant outcomes [19,21]. Our findings support the evidence supporting recommendations of undergoing kidney transplantation before initiating dialysis to improve transplantation outcomes [19,21,22].

There was a wide variability in the dialysis period between the tertile groups. The mean dialysis period in the first tertile was 1.2 months (min-max: 0.5–4), and 21.4 months (min-max: 4–52) in the second tertile, while the third tertile showed a mean dialysis vintage of 107.6 months (min-max: 52–334). There is a consistent shortage of cadaveric donors in Korea with a large proportion of transplantation done with living kidney donors, leading to high number of preemptive or nearly preemptive transplants in the living donor transplant groups, and a considerably longer waitlist time for the cadaveric donor transplant groups. For accurate analysis, we applied several competing risk analysis methods to evaluate the association of pretransplant dialysis vintage with the primary outcomes. In the Cox regression model for all-cause mortality, there was a 3.55-fold increased risk in the third tertile group, while the first and second tertiles showed no significantly increased risk compared to the preemptive transplantation group. In the competing risk regression model for graft failure, there was a 1.89-fold increased risk in the third tertile. There was no significant difference in death-censored graft failure between the preemptive transplantation group and all tertile groups.

To reduce the bias due to the disproportion of dialysis period in different groups, we performed a subanalysis including only DDKT recipients. In the DDKT patients, there was a significant difference in the mean dialysis period between the groups. The mean pretransplant dialysis period of each DDKT tertile group was 41 months (min-max: 0.6–65), 84 months (min-max: 65–102) and 151 months (min-max: 103–334) respectively. Compared with the first tertile, there was a 2.68-fold increase in risk of all-cause mortality in the second tertile, and 3.25-fold increase in the third tertile of the DDKT groups in the Cox-regression analysis. There was no difference in the risk of death-censored graft failure in the DDKT subgroup analysis. Taken together, the DDKT subgroup analysis should be interpreted cautiously. Although longer dialysis vintage was associated with higher all-cause mortality, death-censored graft failure did not differ significantly across tertiles, and the composite outcome did not show a monotonic dose-response pattern. In particular, the composite outcome was significantly higher in the second tertile but not in the third tertile. This suggests that dialysis vintage may not exert a uniform dose-dependent effect on all post-transplant outcomes and may reflect limited statistical power, wide confidence intervals, differences in event composition, or residual confounding within the DDKT subgroup.

The effect of a longer pretransplant dialysis vintage on graft failure or mortality remains incompletely defined in previous studies. Generally, earlier studies reported that a longer pretransplant dialysis duration was associated with higher mortality and increased risk of graft failure [9,17], supporting the need for a shortened waiting time, and waitlist priority to patients on long-term pretransplant dialysis [8,9,23]. However, more recent studies have shown different results regarding the effect of pretransplant dialysis vintage on transplant outcomes. A previous study using the Austrian transplant registry had reported that a longer waiting time on dialysis was not associated with a higher rate of graft loss, but the rate of death was higher in patients on pretransplant dialysis for over 1.5 years compared with patients with dialysis vintage shorter than 1.5 years [12]. In a subgroup analysis, pretransplant dialysis duration did not affect the risk of graft failure in transplants performed after 2000, due to improvements in dialysis and immunosuppression protocols and use of erythropoiesis-stimulating agents which have decreased transfusion frequency, thereby reducing the risk of sensitization [12]. Consistent with these contemporary findings, our results suggest that prolonged dialysis vintage may be more consistently associated with patient mortality than with death-censored graft failure.

In the DDKT recipients, the association between pretransplant dialysis and mortality was significant while the association with death-censored graft loss was less clear. Notably, there was no significant difference in the risk of graft failure between the tertiles in the DDKT patients. These findings are broadly consistent with the Korean cohort study by Lim et al., where the risk of mortality, and composite graft failure and mortality outcome was significantly higher in the third tertile [23]. However, this study reported an increased risk of graft failure in the third tertile, whereas in our study the risk of graft failure was not significantly different between the DDKT tertiles. This discrepancy may be partly explained by differences in cohort composition and immunosuppressive practice. Our cohort had a lower burden of hypertension and diabetes, which may have reduced the contribution of comorbidity-related non-immunologic injury to graft loss. In addition, ATG was used more frequently in our cohort, and BPAR incidence did not differ significantly across dialysis-vintage groups. These findings suggest that differences in immunologic injury may have contributed less prominently to graft failure in our cohort than in previous registry-based data. Therefore, although very long dialysis vintage remained associated with higher mortality, graft survival may have been relatively preserved in this contemporary single-center cohort. Further studies with detailed adjustment for comorbidity burden, immunologic risk, induction strategy, and rejection events are needed to clarify the relationship between dialysis vintage and graft failure.

We have found that a longer pretransplant dialysis was associated with a higher patient mortality. There was a difference in the cause of mortality between the tertile groups. Overall, the most common cause of patient death was from infectious causes. Although death occurred in only 8 patients in the preemptive group, all mortality due to known medical causes were infection-related. 14 deaths occurred in the first tertile, 6 due to infectious causes and 8 due to malignancy. Infection related mortality was highest in the third tertile with 18 deaths, compared with 5, 6 and 4 deaths in the preemptive, 1st and 2nd tertile groups. A previous study using the Finnish registry reported that a longer pretransplant dialysis duration was associated with a higher risk of infectious death, with an adjusted hazard ratio of 1.28 for patients with pretransplant dialysis period over 24 months [24].

Mortality related to cardiovascular events was centered in the third tertile group. Total 14 deaths following MACE occurred in the entire cohort, 12 were in the third tertile group and two were in the second tertile group. No cardiovascular event related mortality was found in the preemptive group and the first tertile group. Previous study by Helanterä et al. reported that a longer duration of pretransplant dialysis was an independent predictor of death from a cardiovascular cause in kidney recipients [23]. However, a more recent study by Goyal et al. [25] had identified age, diabetes, pulmonary circulation disorders and malnutrition as risk factors of MACE, but did not show a clear association with dialysis period. Further studies with a larger number of patients are needed to identify the association between dialysis vintage and cardiovascular risk.

This study has several strengths, including a relatively large single-center cohort with uniform perioperative management and immunosuppressive protocols, a substantial preemptive transplant group rigorously defined as dialysis-naïve, and the use of competing risk models to disentangle the effects of dialysis vintage on graft failure from the competing event of death. The ethnically homogeneous Korean population also adds important data from an Asian setting, complementing the predominantly Western literature.

Several limitations should be acknowledged. First, the retrospective, observational design precludes causal inference, and residual confounding by unmeasured factors, such as frailty, functional and socioeconomic status, dialysis adequacy, treatment adherence, and cardiovascular risk burden, cannot be excluded. Second, the distribution of dialysis duration differed substantially between living and deceased donor recipients, reflecting Korean donor availability and allocation practices but complicating direct comparisons; the DDKT subgroup analysis partly addresses this but reduces statistical power. Third, although the study period reflects contemporary transplant practice, changes in immunosuppressive strategies, perioperative care, and dialysis management over time may have influenced outcomes. Fourth, categorizing dialysis vintage into tertiles, while pragmatic, may mask non-linear relationships and does not capture time-varying effects. Because the tertile boundaries were cohort-derived, they should be interpreted as exploratory risk strata and require validation in independent cohorts. Finally, the findings from a single tertiary center may not be fully generalizable to other healthcare systems or ethnic groups. Future multicenter or registry-based studies using predefined clinical cutoffs, continuous modeling, or spline-based analyses are needed to determine whether a reproducible risk threshold exists.

In conclusion, a longer period of pretransplant dialysis was associated with a higher risk of all-cause mortality and cardiovascular event related mortality, while the association with graft failure was unclear. Earlier kidney transplantation and reduced waitlist time is beneficial for reducing the long-term mortality of ESRD patients.

## Supporting information

S1 Table Forest plot for subgroup analyses for all-cause mortality. (DOCX)

S2 Table Forest plot for subgroup analyses for death-censored graft failure. (DOCX)

S3 Table Forest plot for subgroup analyses for composite outcome (both all-cause mortality and death-censored graft failure). (DOCX)

S1 Data Supplementary file data. Original data. (DOCX)

## Abbreviations

KT: Kidney transplantation
ESRD: End-stage renal disease
LDKT: Living donor kidney transplantation
DDKT: Deceased donor kidney transplantation
MACE: Major adverse cardiovascular events
BPAR: Biopsy-proven acute rejection
DGF: Delayed graft function
ATG: Antithymocyte globulin
IQR: Interquartile range
ARDS: Acute respiratory distress syndrome
HR: Hazard ratio
CI: Confidence interval
aHR: Adjusted hazard ratio
DM: Diabetes mellitus
HTN: Hypertension
