# Supplementary material for: Pre-transplant dialysis vintage and post-transplant outcomes: A retrospective cohort study in Korean kidney transplant recipients
Source: PLoS One. 2026 Jul 20;21(7):e0352995. doi: 10.1371/journal.pone.0352995 (PMC13384295; doi:10.1371/journal.pone.0352995)
Supplement: S1 Table — (DOCX) [file pone.0352995.s001.docx]

S1 Table. Forest plot for subgroup analyses for all-cause mortality

|  |  | Vintage group | No event | event | aHR (95% CI) | *p* value |
| --- | --- | --- | --- | --- | --- | --- |
| Recipient age | ≤50 | Preemptive | 197 (97.0) | 6 (3.0) |  |  |
|  |  | Tertile 1 | 308 (99.0) | 3 (1.0) | 0.31(0.08-1.24) | 0.098 |
|  |  | Tertile 2 | 273 (98.9) | 3 (1.1) | 0.32(0.08-1.24) | 0.100 |
|  |  | Tertile 3 | 238 (94.8) | 13 (5.2) | 1.12(0.40-3.14) | 0.831 |
|  | >50 | Preemptive | 164 (98.8) | 2 (1.2) |  |  |
|  |  | Tertile 1 | 169 (93.4) | 12 (6.6) | 4.15(1.02-16.81) | 0.046 |
|  |  | Tertile 2 | 202 (94.0) | 13 (6.0) | 3.31(0.82-13.34) | 0.092 |
|  |  | Tertile 3 | 204 (85.0) | 36 (15.0) | 9.00(2.39-33.93) | 0.001 |
| Sex | Male | Preemptive | 207 (97.6) | 5 (2.4) |  |  |
|  |  | Tertile 1 | 284 (95.6) | 13 (4.4) | 1.47(0.52-4.12) | 0.464 |
|  |  | Tertile 2 | 282 (96.2) | 11 (3.8) | 1.08(0.38-3.09) | 0.888 |
|  |  | Tertile 3 | 240 (87.9) | 33 (12.1) | 3.59(1.40-9.21) | 0.008 |
|  | Female | Preemptive | 154 (98.1) | 3 (1.9) |  |  |
|  |  | Tertile 1 | 193 (99.0) | 2 (1.0) | 0.63(0.11-3.51) | 0.599 |
|  |  | Tertile 2 | 193 (97.5) | 5 (2.5) | 1.00(0.24-4.14) | 0.999 |
|  |  | Tertile 3 | 202 (92.7) | 16 (7.3) | 1.87(0.54-6.50) | 0.328 |
| BMI | ≤25 | Preemptive | 261 (97.4) | 7 (2.6) |  |  |
|  |  | Tertile 1 | 340 (98.0) | 7 (2.0) | 0.73(0.26-2.08) | 0.553 |
|  |  | Tertile 2 | 342 (97.2) | 10 (2.8) | 0.80(0.30-2.10) | 0.649 |
|  |  | Tertile 3 | 327 (90.3) | 35 (9.7) | 2.47(1.09-5.62) | 0.031 |
|  | >25 | Preemptive | 100 (99.0) | 1 (1.0) |  |  |
|  |  | Tertile 1 | 137 (94.5) | 8 (5.5) | 3.95(0.61-25.54) | 0.150 |
|  |  | Tertile 2 | 133 (95.7) | 6 (4.3) | 2.83(0.43-18.49) | 0.277 |
|  |  | Tertile 3 | 115 (89.1) | 14 (10.9) | 4.86(0.76-30.94) | 0.094 |
| DM | No | Preemptive | 258 (97.7) | 6 (2.3) |  |  |
|  |  | Tertile 1 | 329 (97.6) | 8 (2.4) | 1.02(0.36-2.94) | 0.964 |
|  |  | Tertile 2 | 317 (97.5) | 8 (2.5) | 0.84(0.29-2.41) | 0.748 |
|  |  | Tertile 3 | 355 (91.7) | 32 (8.3) | 1.93(0.80-4.66) | 0.143 |
|  | Yes | Preemptive | 103 (98.1) | 2 (1.9) |  |  |
|  |  | Tertile 1 | 148 (95.5) | 7 (4.5) | 2.10(0.46-9.56) | 0.338 |
|  |  | Tertile 2 | 158 (95.2) | 8 (4.8) | 1.98(0.45-8.83) | 0.369 |
|  |  | Tertile 3 | 87 (83.7) | 17 (16.3) | 7.01(1.72-28.62) | 0.007 |
| DDKT | No | Preemptive | 47 (95.9) | 2 (4.1) |  |  |
|  |  | Tertile 1 | 66 (98.5) | 1 (1.5) | 0.30(0.03-3.05) | 0.306 |
|  |  | Tertile 2 | 82 (94.3) | 5 (5.7) | 0.65(0.11-3.94) | 0.640 |
|  |  | Tertile 3 | 74 (92.5) | 6 (7.5) | 0.65(0.11-3.76) | 0.629 |
|  | Yes | Preemptive | 314 (98.1) | 6 (1.9) |  |  |
|  |  | Tertile 1 | 411 (96.7) | 14 (3.3) | 1.69(0.65-4.35) | 0.279 |
|  |  | Tertile 2 | 393 (97.3) | 11 (2.7) | 1.16(0.44-3.10) | 0.763 |
|  |  | Tertile 3 | 368 (89.5) | 43 (10.5) | 4.09(1.74-9.58) | 0.001 |
| Number of plasmapheresis | <3 | Preemptive | 359 (97.8) | 8 (2.2) |  |  |
|  |  | Tertile 1 | 471 (96.9) | 15 (3.1) | 1.29(0.54-3.07) | 0.571 |
|  |  | Tertile 2 | 377 (96.7) | 13 (3.3) | 1.10(0.45-2.71) | 0.829 |
|  |  | Tertile 3 | 86 (89.6) | 10 (10.4) | 3.51(1.33-9.26) | 0.011 |
|  | ≥3 | Preemptive | 2 (100.0) | 0 (0.0) | N/A |  |
|  |  | Tertile 1 | 6 (100.0) | 0 (0.0) | N/A |  |
|  |  | Tertile 2 | 98 (97.0) | 3 (3.0) | reference |  |
|  |  | Tertile 3 | 356 (90.1) | 39 (9.9) | 3.08(1.01-9.44) | 0.049 |
| Induction agent | Basiliximab | Preemptive | 271 (99.3) | 2 (0.7) |  |  |
|  |  | Tertile 1 | 358 (96.2) | 14 (3.8) | 3.93(1.00-15.48) | 0.050 |
|  |  | Tertile 2 | 368 (96.6) | 13 (3.4) | 2.91(0.74-11.49) | 0.127 |
|  |  | Tertile 3 | 423 (90.0) | 47 (10.0) | 7.82(2.13-28.75) | 0.002 |
|  | ATG | Preemptive | 90 (93.8) | 6 (6.3) |  |  |
|  |  | Tertile 1 | 119 (99.2) | 1 (0.8) | 0.10(0.01-0.89) | 0.039 |
|  |  | Tertile 2 | 107 (97.3) | 3 (2.7) | 0.23(0.04-1.26) | 0.090 |
|  |  | Tertile 3 | 19 (90.5) | 2 (9.5) | 1.85(0.34-9.99) | 0.474 |
| KT year | <2010 | Preemptive | 301 (98.0) | 6 (2.0) |  |  |
|  |  | Tertile 1 | 411 (96.7) | 14 (3.3) | 1.48(0.57-3.83) | 0.422 |
|  |  | Tertile 2 | 381 (96.5) | 14 (3.5) | 1.37(0.53-3.57) | 0.514 |
|  |  | Tertile 3 | 297 (90.0) | 33 (10.0) | 3.34(1.38-8.06) | 0.007 |
|  | ≥2010 | Preemptive | 60 (96.8) | 2 (3.2) |  |  |
|  |  | Tertile 1 | 66 (98.5) | 1 (1.5) | 0.54(0.06-4.94) | 0.582 |
|  |  | Tertile 2 | 94 (97.9) | 2 (2.1) | 0.45(0.07-2.88) | 0.396 |
|  |  | Tertile 3 | 145 (90.1) | 16 (9.9) | 1.39(0.32-6.11) | 0.661 |
| Donor age | ≤48 | Preemptive | 24 (100.0) | 0 (0.0) |  |  |
|  |  | Tertile 1 | 63 (92.6) | 5 (7.4) | 3.78(0.17-85.84) | 0.403 |
|  |  | Tertile 2 | 67 (95.7) | 3 (4.3) | 2.14(0.09-52.43) | 0.641 |
|  |  | Tertile 3 | 62 (89.9) | 7 (10.1) | 3.02(0.14-66.60) | 0.484 |
|  | >48 | Preemptive | 337 (97.7) | 8 (2.3) |  |  |
|  |  | Tertile 1 | 414 (97.6) | 10 (2.4) | 1.03(0.41-2.61) | 0.943 |
|  |  | Tertile 2 | 408 (96.9) | 13 (3.1) | 1.02(0.42-2.47) | 0.960 |
|  |  | Tertile 3 | 380 (90.0) | 42 (10.0) | 3.07(1.43-6.60) | 0.004 |
| Donor BMI | ≤25 | Preemptive | 194 (97.5) | 5 (2.5) |  |  |
|  |  | Tertile 1 | 281 (97.9) | 6 (2.1) | 0.81(0.25-2.64) | 0.724 |
|  |  | Tertile 2 | 271 (97.1) | 8 (2.9) | 0.94(0.31-2.87) | 0.917 |
|  |  | Tertile 3 | 232 (90.6) | 24 (9.4) | 2.83(1.06-7.53) | 0.038 |
|  | >25 | Preemptive | 167 (98.2) | 3 (1.8) |  |  |
|  |  | Tertile 1 | 196 (95.6) | 9 (4.4) | 2.00(0.56-7.21) | 0.288 |
|  |  | Tertile 2 | 204 (96.2) | 8 (3.8) | 1.28(0.35-4.74) | 0.713 |
|  |  | Tertile 3 | 209 (90.1) | 23 (9.9) | 3.32(1.01-10.93) | 0.048 |

Adjusted for recipient age, DM, HTN, primary renal diagnosis, mismatch number, transplantation year and donor age. aHR, adjusted hazard ratio; CI, confidence interval; BMI, body mass index; DM, diabetes mellitus; DDKT, deceased donor kidney transplantation; ATG, anti-thymocyte globulin; KT, kidney transplantation
