# Supplementary material for: Pre-transplant dialysis vintage and post-transplant outcomes: A retrospective cohort study in Korean kidney transplant recipients
Source: PLoS One. 2026 Jul 20;21(7):e0352995. doi: 10.1371/journal.pone.0352995 (PMC13384295; doi:10.1371/journal.pone.0352995)
Supplement: S2 Table — (DOCX) [file pone.0352995.s002.docx]

S2 Table. Forest plot for subgroup analyses for death-censored graft failure

|  |  | Vintage group | No event | event | aHR (95% CI) | *p* value |
| --- | --- | --- | --- | --- | --- | --- |
| Recipient age | ≤50 | Preemptive | 179 (88.2) | 24 (11.8) |  |  |
|  |  | Tertile 1 | 274 (88.1) | 37 (11.9) | 0.91(0.52-1.57) | 0.725 |
|  |  | Tertile 2 | 231 (83.7) | 45 (16.3) | 1.29(0.76-2.17) | 0.346 |
|  |  | Tertile 3 | 205 (81.7) | 46 (18.3) | 1.01(0.57-1.78) | 0.977 |
|  | >50 | Preemptive | 156 (94.0) | 10 (6.0) |  |  |
|  |  | Tertile 1 | 154 (85.1) | 27 (14.9) | 1.96(0.86-4.43) | 0.108 |
|  |  | Tertile 2 | 185 (86.0) | 30 (14.0) | 1.68(0.74-3.77) | 0.212 |
|  |  | Tertile 3 | 174 (72.5) | 66 (27.5) | 3.39(1.58-7.28) | 0.002 |
| Sex | Male | Preemptive | 194 (91.5) | 18 (8.5) |  |  |
|  |  | Tertile 1 | 254 (85.5) | 43 (14.5) | 1.45(0.78-2.69) | 0.235 |
|  |  | Tertile 2 | 245 (83.6) | 48 (16.4) | 1.76(0.97-3.18) | 0.063 |
|  |  | Tertile 3 | 205 (75.1) | 68 (24.9) | 2.13(1.17-3.89) | 0.013 |
|  | Female | Preemptive | 141 (89.8) | 16 (10.2) |  |  |
|  |  | Tertile 1 | 174 (89.2) | 21 (10.8) | 0.93(0.47-1.83) | 0.825 |
|  |  | Tertile 2 | 171 (86.4) | 27 (13.6) | 1.11(0.58-2.12) | 0.752 |
|  |  | Tertile 3 | 174 (79.8) | 44 (20.2) | 1.35(0.71-2.54) | 0.36 |
| BMI | ≤25 | Preemptive | 240 (89.6) | 28 (10.4) |  |  |
|  |  | Tertile 1 | 310 (89.3) | 37 (10.7) | 0.86(0.51-1.45) | 0.575 |
|  |  | Tertile 2 | 302 (85.8) | 50 (14.2) | 1.12(0.69-1.83) | 0.651 |
|  |  | Tertile 3 | 280 (77.3) | 82 (22.7) | 1.47(0.91-2.38) | 0.119 |
|  | >25 | Preemptive | 95 (94.1) | 6 (5.9) |  |  |
|  |  | Tertile 1 | 118 (81.4) | 27 (18.6) | 2.54(0.91-7.06) | 0.074 |
|  |  | Tertile 2 | 114 (82.0) | 25 (18.0) | 2.70(0.99-7.39) | 0.053 |
|  |  | Tertile 3 | 99 (76.7) | 30 (23.3) | 2.26(0.82-6.25) | 0.116 |
| DM | No | Preemptive | 240 (90.9) | 24 (9.1) |  |  |
|  |  | Tertile 1 | 300 (89.0) | 37 (11.0) | 1.00(0.57-1.74) | 0.996 |
|  |  | Tertile 2 | 281 (86.5) | 44 (13.5) | 1.28(0.75-2.19) | 0.361 |
|  |  | Tertile 3 | 307 (79.3) | 80 (20.7) | 1.53(0.92-2.55) | 0.103 |
|  | Yes | Preemptive | 95 (90.5) | 10 (9.5) |  |  |
|  |  | Tertile 1 | 128 (82.6) | 27 (17.4) | 1.60(0.73-3.54) | 0.244 |
|  |  | Tertile 2 | 135 (81.3) | 31 (18.7) | 1.81(0.85-3.84) | 0.124 |
|  |  | Tertile 3 | 72 (69.2) | 32 (30.8) | 2.05(0.95-4.41) | 0.067 |
| DDKT | No | Preemptive | 40 (81.6) | 9 (18.4) |  |  |
|  |  | Tertile 1 | 61 (91.0) | 6 (9.0) | 0.30(0.09-0.97) | 0.045 |
|  |  | Tertile 2 | 70 (80.5) | 17 (19.5) | 1.03(0.44-2.37) | 0.952 |
|  |  | Tertile 3 | 66 (82.5) | 14 (17.5) | 0.71(0.30-1.69) | 0.439 |
|  | Yes | Preemptive | 295 (92.2) | 25 (7.8) |  |  |
|  |  | Tertile 1 | 367 (86.4) | 58 (13.6) | 1.59(0.95-2.69) | 0.08 |
|  |  | Tertile 2 | 346 (85.6) | 58 (14.4) | 1.64(0.97-2.76) | 0.065 |
|  |  | Tertile 3 | 313 (76.2) | 98 (23.8) | 2.09(1.25-3.48) | 0.005 |
| Number of plasmapheresis | <3 | Preemptive | 334 (91.0) | 33 (9.0) |  |  |
|  |  | Tertile 1 | 424 (87.2) | 62 (12.8) | 1.13(0.71-1.81) | 0.595 |
|  |  | Tertile 2 | 331 (84.9) | 59 (15.1) | 1.42(0.89-2.26) | 0.141 |
|  |  | Tertile 3 | 71 (74.0) | 25 (26.0) | 2.29(1.26-4.15) | 0.006 |
|  | ≥3 | Preemptive | 1 (50.0) | 1 (50.0) |  |  |
|  |  | Tertile 1 | 4 (66.7) | 2 (33.3) | 0.68(0.04-10.55) | 0.779 |
|  |  | Tertile 2 | 85 (84.2) | 16 (15.8) | 0.28(0.03-2.77) | 0.278 |
|  |  | Tertile 3 | 308 (78.0) | 87 (22.0) | 0.32(0.03-3.05) | 0.325 |
| Induction agent | Basiliximab | Preemptive | 250 (91.6) | 23 (8.4) |  |  |
|  |  | Tertile 1 | 318 (85.5) | 54 (14.5) | 1.19(0.71-1.97) | 0.514 |
|  |  | Tertile 2 | 327 (85.8) | 54 (14.2) | 1.30(0.79-2.14) | 0.299 |
|  |  | Tertile 3 | 362 (77.0) | 108 (23.0) | 1.73(1.07-2.80) | 0.024 |
|  | ATG | Preemptive | 85 (88.5) | 11 (11.5) |  |  |
|  |  | Tertile 1 | 110 (91.7) | 10 (8.3) | 1.31(0.47-3.60) | 0.606 |
|  |  | Tertile 2 | 89 (80.9) | 21 (19.1) | 1.86(0.71-4.92) | 0.209 |
|  |  | Tertile 3 | 17 (81.0) | 4 (19.0) | 1.85(0.43-7.92) | 0.41 |
| KT year | <2010 | Preemptive | 277 (90.2) | 30 (9.8) |  |  |
|  |  | Tertile 1 | 369 (86.8) | 56 (13.2) | 1.08(0.67-1.74) | 0.763 |
|  |  | Tertile 2 | 330 (83.5) | 65 (16.5) | 1.40(0.88-2.22) | 0.156 |
|  |  | Tertile 3 | 249 (75.5) | 81 (24.5) | 1.64(1.02-2.64) | 0.04 |
|  | ≥2010 | Preemptive | 58 (93.5) | 4 (6.5) |  |  |
|  |  | Tertile 1 | 59 (88.1) | 8 (11.9) | 2.32(0.59-9.18) | 0.229 |
|  |  | Tertile 2 | 86 (89.6) | 10 (10.4) | 1.61(0.43-6.01) | 0.481 |
|  |  | Tertile 3 | 130 (80.7) | 31 (19.3) | 1.83(0.54-6.15) | 0.33 |
| Donor age | ≤48 | Preemptive | 15 (62.5) | 9 (37.5) |  |  |
|  |  | Tertile 1 | 52 (76.5) | 16 (23.5) | 0.43(0.19-0.96) | 0.038 |
|  |  | Tertile 2 | 48 (68.6) | 22 (31.4) | 0.84(0.38-1.84) | 0.659 |
|  |  | Tertile 3 | 45 (65.2) | 24 (34.8) | 0.70(0.30-1.60) | 0.392 |
|  | >48 | Preemptive | 320 (92.8) | 25 (7.2) |  |  |
|  |  | Tertile 1 | 376 (88.7) | 48 (11.3) | 1.52(0.89-2.61) | 0.124 |
|  |  | Tertile 2 | 368 (87.4) | 53 (12.6) | 1.61(0.95-2.73) | 0.08 |
|  |  | Tertile 3 | 334 (79.1) | 88 (20.9) | 2.18(1.30-3.63) | 0.003 |
| Donor BMI | ≤25 | Preemptive | 177 (88.9) | 22 (11.1) |  |  |
|  |  | Tertile 1 | 251 (87.5) | 36 (12.5) | 1.01(0.57-1.79) | 0.964 |
|  |  | Tertile 2 | 238 (85.3) | 41 (14.7) | 1.23(0.72-2.11) | 0.456 |
|  |  | Tertile 3 | 203 (79.3) | 53 (20.7) | 1.30(0.75-2.25) | 0.35 |
|  | >25 | Preemptive | 158 (92.9) | 12 (7.1) |  |  |
|  |  | Tertile 1 | 177 (86.3) | 28 (13.7) | 1.41(0.67-2.98) | 0.368 |
|  |  | Tertile 2 | 178 (84.0) | 34 (16.0) | 1.73(0.83-3.60) | 0.141 |
|  |  | Tertile 3 | 175 (75.4) | 57 (24.6) | 2.11(1.03-4.32) | 0.042 |

Adjusted for recipient age, DM, HTN, primary renal diagnosis, mismatch number, transplantation year and donor age. aHR, adjusted hazard ratio; CI, confidence interval; BMI, body mass index; DM, diabetes mellitus; DDKT, deceased donor kidney transplantation; ATG, anti-thymocyte globulin; KT, kidney transplantation
