# Supplementary material for: Pre-transplant dialysis vintage and post-transplant outcomes: A retrospective cohort study in Korean kidney transplant recipients
Source: PLoS One. 2026 Jul 20;21(7):e0352995. doi: 10.1371/journal.pone.0352995 (PMC13384295; doi:10.1371/journal.pone.0352995)
Supplement: S3 Table — (DOCX) [file pone.0352995.s003.docx]

S3 Table. Forest plot for subgroup analyses for composite outcome (both all-cause mortality and death-censored graft failure)

|  |  | Vintage group | No event | event | aHR (95% CI) | *p* value |
| --- | --- | --- | --- | --- | --- | --- |
| Recipient age | ≤50 | Preemptive | 179 (88.2) | 24 (11.8) |  |  |
|  |  | Tertile 1 | 274 (88.1) | 37 (11.9) | 0.83(0.49-1.38) | 0.467 |
|  |  | Tertile 2 | 231 (83.7) | 45 (16.3) | 1.09(0.66-1.79) | 0.736 |
|  |  | Tertile 3 | 205 (81.7) | 46 (18.3) | 1.11(0.67-1.84) | 0.693 |
|  | >50 | Preemptive | 156 (94.0) | 10 (6.0) |  |  |
|  |  | Tertile 1 | 154 (85.1) | 27 (14.9) | 2.22(1.08-4.58) | 0.031 |
|  |  | Tertile 2 | 185 (86.0) | 30 (14.0) | 1.75(0.85-3.59) | 0.126 |
|  |  | Tertile 3 | 174 (72.5) | 66 (27.5) | 3.95(2.02-7.71) | <0.001 |
| Sex | Male | Preemptive | 194 (91.5) | 18 (8.5) |  |  |
|  |  | Tertile 1 | 254 (85.5) | 43 (14.5) | 1.44(0.83-2.50) | 0.199 |
|  |  | Tertile 2 | 245 (83.6) | 48 (16.4) | 1.44(0.83-2.48) | 0.192 |
|  |  | Tertile 3 | 205 (75.1) | 68 (24.9) | 2.41(1.42-4.08) | 0.001 |
|  | Female | Preemptive | 141 (89.8) | 16 (10.2) |  |  |
|  |  | Tertile 1 | 174 (89.2) | 21 (10.8) | 0.93(0.48-1.79) | 0.83 |
|  |  | Tertile 2 | 171 (86.4) | 27 (13.6) | 1.15(0.62-2.13) | 0.656 |
|  |  | Tertile 3 | 174 (79.8) | 44 (20.2) | 1.46(0.81-2.64) | 0.214 |
| BMI | ≤25 | Preemptive | 240 (89.6) | 28 (10.4) |  |  |
|  |  | Tertile 1 | 310 (89.3) | 37 (10.7) | 0.85(0.52-1.40) | 0.528 |
|  |  | Tertile 2 | 302 (85.8) | 50 (14.2) | 1.03(0.64-1.64) | 0.915 |
|  |  | Tertile 3 | 280 (77.3) | 82 (22.7) | 1.67(1.07-2.59) | 0.023 |
|  | >25 | Preemptive | 95 (94.1) | 6 (5.9) |  |  |
|  |  | Tertile 1 | 118 (81.4) | 27 (18.6) | 2.65(1.10-6.42) | 0.03 |
|  |  | Tertile 2 | 114 (82.0) | 25 (18.0) | 2.43(1.01-5.86) | 0.048 |
|  |  | Tertile 3 | 99 (76.7) | 30 (23.3) | 2.57(1.05-6.26) | 0.038 |
| DM | No | Preemptive | 240 (90.9) | 24 (9.1) |  |  |
|  |  | Tertile 1 | 300 (89.0) | 37 (11.0) | 1.02(0.61-1.71) | 0.948 |
|  |  | Tertile 2 | 281 (86.5) | 44 (13.5) | 1.18(0.72-1.95) | 0.511 |
|  |  | Tertile 3 | 307 (79.3) | 80 (20.7) | 1.61(1.01-2.58) | 0.046 |
|  | Yes | Preemptive | 95 (90.5) | 10 (9.5) |  |  |
|  |  | Tertile 1 | 128 (82.6) | 27 (17.4) | 1.71(0.83-3.55) | 0.149 |
|  |  | Tertile 2 | 135 (81.3) | 31 (18.7) | 1.68(0.82-3.43) | 0.155 |
|  |  | Tertile 3 | 72 (69.2) | 32 (30.8) | 2.85(1.39-5.81) | 0.004 |
| DDKT | No | Preemptive | 40 (81.6) | 9 (18.4) |  |  |
|  |  | Tertile 1 | 61 (91.0) | 6 (9.0) | 0.36(0.13-1.05) | 0.062 |
|  |  | Tertile 2 | 70 (80.5) | 17 (19.5) | 0.96(0.39-2.34) | 0.922 |
|  |  | Tertile 3 | 66 (82.5) | 14 (17.5) | 0.68(0.27-1.74) | 0.426 |
|  | Yes | Preemptive | 295 (92.2) | 25 (7.8) |  |  |
|  |  | Tertile 1 | 367 (86.4) | 58 (13.6) | 1.57(0.98-2.51) | 0.062 |
|  |  | Tertile 2 | 346 (85.6) | 58 (14.4) | 1.45(0.90-2.32) | 0.123 |
|  |  | Tertile 3 | 313 (76.2) | 98 (23.8) | 2.41(1.54-3.76) | <0.001 |
| Number of plasmapheresis | <3 | Preemptive | 334 (91.0) | 33 (9.0) |  |  |
|  |  | Tertile 1 | 424 (87.2) | 62 (12.8) | 1.19(0.78-1.83) | 0.415 |
|  |  | Tertile 2 | 331 (84.9) | 59 (15.1) | 1.34(0.87-2.07) | 0.179 |
|  |  | Tertile 3 | 71 (74.0) | 25 (26.0) | 2.49(1.46-4.23) | 0.001 |
|  | ≥3 | Preemptive | 1 (50.0) | 1 (50.0) |  |  |
|  |  | Tertile 1 | 4 (66.7) | 2 (33.3) | 0.50(0.06-4.27) | 0.527 |
|  |  | Tertile 2 | 85 (84.2) | 16 (15.8) | 0.17(0.03-0.99) | 0.049 |
|  |  | Tertile 3 | 308 (78.0) | 87 (22.0) | 0.27(0.05-1.49) | 0.133 |
| Induction agent | Basiliximab | Preemptive | 250 (91.6) | 23 (8.4) |  |  |
|  |  | Tertile 1 | 318 (85.5) | 54 (14.5) | 1.44(0.88-2.35) | 0.145 |
|  |  | Tertile 2 | 327 (85.8) | 54 (14.2) | 1.32(0.81-2.16) | 0.261 |
|  |  | Tertile 3 | 362 (77.0) | 108 (23.0) | 2.22(1.40-3.51) | 0.001 |
|  | ATG | Preemptive | 85 (88.5) | 11 (11.5) |  |  |
|  |  | Tertile 1 | 110 (91.7) | 10 (8.3) | 0.85(0.35-2.03) | 0.711 |
|  |  | Tertile 2 | 89 (80.9) | 21 (19.1) | 1.39(0.64-3.03) | 0.407 |
|  |  | Tertile 3 | 17 (81.0) | 4 (19.0) | 1.84(0.58-5.79) | 0.299 |
| KT year | <2010 | Preemptive | 277 (90.2) | 30 (9.8) |  |  |
|  |  | Tertile 1 | 369 (86.8) | 56 (13.2) | 1.14(0.73-1.79) | 0.554 |
|  |  | Tertile 2 | 330 (83.5) | 65 (16.5) | 1.32(0.86-2.05) | 0.209 |
|  |  | Tertile 3 | 249 (75.5) | 81 (24.5) | 1.83(1.19-2.81) | 0.006 |
|  | ≥2010 | Preemptive | 58 (93.5) | 4 (6.5) |  |  |
|  |  | Tertile 1 | 59 (88.1) | 8 (11.9) | 1.87(0.57-6.11) | 0.302 |
|  |  | Tertile 2 | 86 (89.6) | 10 (10.4) | 1.15(0.37-3.60) | 0.807 |
|  |  | Tertile 3 | 130 (80.7) | 31 (19.3) | 1.87(0.66-5.26) | 0.237 |
| Donor age | ≤48 | Preemptive | 15 (62.5) | 9 (37.5) |  |  |
|  |  | Tertile 1 | 52 (76.5) | 16 (23.5) | 0.51(0.22-1.16) | 0.109 |
|  |  | Tertile 2 | 48 (68.6) | 22 (31.4) | 0.84(0.38-1.84) | 0.659 |
|  |  | Tertile 3 | 45 (65.2) | 24 (34.8) | 0.81(0.36-1.83) | 0.609 |
|  | >48 | Preemptive | 320 (92.8) | 25 (7.2) |  |  |
|  |  | Tertile 1 | 376 (88.7) | 48 (11.3) | 1.49(0.92-2.43) | 0.104 |
|  |  | Tertile 2 | 368 (87.4) | 53 (12.6) | 1.43(0.88-2.30) | 0.145 |
|  |  | Tertile 3 | 334 (79.1) | 88 (20.9) | 2.42(1.54-3.80) | <0.001 |
| Donor BMI | ≤25 | Preemptive | 177 (88.9) | 22 (11.1) |  |  |
|  |  | Tertile 1 | 251 (87.5) | 36 (12.5) | 1.02(0.60-1.74) | 0.948 |
|  |  | Tertile 2 | 238 (85.3) | 41 (14.7) | 1.16(0.69-1.96) | 0.574 |
|  |  | Tertile 3 | 203 (79.3) | 53 (20.7) | 1.59(0.95-2.64) | 0.075 |
|  | >25 | Preemptive | 158 (92.9) | 12 (7.1) |  |  |
|  |  | Tertile 1 | 177 (86.3) | 28 (13.7) | 1.53(0.78-3.02) | 0.218 |
|  |  | Tertile 2 | 178 (84.0) | 34 (16.0) | 1.55(0.80-3.00) | 0.198 |
|  |  | Tertile 3 | 175 (75.4) | 57 (24.6) | 2.29(1.21-4.32) | 0.01 |

Adjusted for recipient age, DM, HTN, primary renal diagnosis, mismatch number, transplantation year and donor age. aHR, adjusted hazard ratio; CI, confidence interval; BMI, body mass index; DM, diabetes mellitus; DDKT, deceased donor kidney transplantation; ATG, anti-thymocyte globulin; KT, kidney transplantation
